# Supplementary material for: Coronavirus disease 2019 (COVID-19) vaccine acceptability in Ghana: An urban-based population study
Source: PLoS One. 2025 Mar 18;20(3):e0319798. doi: 10.1371/journal.pone.0319798 (PMC11918374; doi:10.1371/journal.pone.0319798)
Supplement: Attachment — Submitted filename: Response to reviewers_2023.08.11.docx [file pone.0319798.s002.docx]

| Response to reviewers comments: Manuscript number PONE-D-23-13433  “Coronavirus disease 2019 (COVID-19) vaccine acceptability in Ghana: An urban-based population study” | |
| --- | --- |
| **Additional Editor Comments** | **Authors’ Response to comments** |
| Please amend your manuscript to include your abstract after the title page. | The manuscript has been amended and the abstract has been inserted after the tile page |
|  |  |
| This manuscript required major revision and I encourage the authors to address point by point of reviewer comments and submit the updated version of the manuscript for further evaluation. | The manuscript has been extensively revised to address the editor’s and reviewers’ comments. |
|  |  |
| **Reviewer #1’s comments** | **Authors’ Response to comments** |
| This manuscript needs significant improvement. The study rationale is poorly articulated and doesn’t answer the “So What” question. | The authors have revised the manuscript extensively. This includes the introduction section of the manuscript to reflect the rationale of the study and the implications for promoting health in the study setting. |
| **Abstract** | Authors acknowledge your valuable comments. The abstract has been revised to reflect the comments made by the reviewer in the manuscript. |
| The study site and the time of the study are missing. How the study was implemented is missing. How the respondents received the questionnaires?  **Results:** The proportion could be presented and the numerator and denominator could be presented in parenthesis. The denominator should be mentioned here. Vaccine hesitancy 301 (80%) -what is the denominator here. “Out of the 377 participants, their Socio-demographic characteristics showed that the majority were below 25 years (203, 53.8%) [vaccine acceptance; 28(13.8%) vs vaccine hesitancy; 175(86.2%)], and Females (189, 50.1%) [vaccine acceptance; 43(22.8%) vs vaccine hesitancy; 146(77.2%)].” The way results are presented is difficult to understand.  Mistrust, Personal- the capital letter should be avoided.  Conclusion “COVID-19 vaccine acceptance was low with high vaccine hesitancy among participants”. It’s already known. What new knowledge this paper is going to add to the literature. Did you find any factors that mostly contributing to this low acceptance. | **Method**  The method section has been revised to include the study site and date it was conducted, together with how the data was collected.  The revised section read  “A cross sectional study was conducted between September and November, 2022 using a multi-stage cluster sampling procedure among 377 participants. A modified World Health Organization pretested paper-based questionnaire was administered to study participants.”  This text can be found on **page 2 Line 32-34**  **Results**  The denominator has been included in the narration of the results of the study.  The revised section read: “The study revealed that COVID-19 vaccine acceptance was 20.0% (76/377) and vaccine hesitancy was 80.0% (301/377).  Out of the 377 participants, their socio-demographic characteristics showed that the majority were below 25 years 53.8% (203/377), [vaccine acceptance; 36.84% (28/76) vs vaccine hesitancy; 58.14% (175/301)], and females 50.1% (189/377), [vaccine acceptance; 56.58% (43/76) vs vaccine hesitancy; 48.50% (146/301)]..”  The revised text can be found **on Page 2 Line 39-43**  The capital letters have been removed.  **Page 2 :Line 44-45**  **Conclusion**  The conclusion has been revised to highlight the most important findings and its implications to vaccine hesitancy/low vaccine acceptance in similar settings. The revised version read:  “The study’s findings highlights the importance of addressing vaccine hesitancy through building trust in the vaccine development processes, including the provision of accurate information about the vaccine safety and efficacy. Resolving concerns related to the source of the vaccine and the overall healthcare system are important to address vaccine hesitancy. Policy makers could adopt tailored interventions targeting specific demographic groups, such as the younger population and females to increase vaccine acceptance.”  The text can be found on **Page 2 Line 50-56** |
| **Manuscript** |  |
| Introduction  This manuscript needs significant improvement. The study rationale is poorly articulated and doesn’t answer the “So What” question. | The introduction has equally been revised to address the shortfalls. New text have been inserted and highlighted. Please they can be found on **Page 5 Line 92-98; 103-109** |
| Methods Data collection and procedures “The questionnaires were interviewer assisted and respondents were assured that their participation was entirely voluntary”. In the abstract you have mentioned self-administered questionnaire. | Authors acknowledge your valuable comments.  The statement “respondents were assured that their participation was entirely voluntary” was to reinforce the ethical considerations employed in the study. Authors have therefore moved the statement to the ethical section of the study.  Also, authors have revised the statement on the data collection process.  Again, the interviewer assistance approached was used to assist persons who could not read the English language whereas the self-administered was used for individuals who could read.  Authors have revised the statements to bring clarity.  The revise statement now read:  “Respondents, who could not read English, were assisted by the data enumerators, and those who could read attempted the questions themselves.” The text can be found on **Page 8 lines 160-162** |
| **Processing and statistical analysis**  “Finally, Multivariate Logistic Regression Model was performed with selected variables that had significant relationship at the chi-square level with odds ratios (OR) and their 95% confidence intervals (95%CI). A p-value of less than 0.05 was considered statistically significant”. What criteria was followed to select variables for the multivariate model?  “Had significant relationship at the chi-square level with odds ratios (OR)” not sure what do you mean by that. Do you mean the variable was significant in both chi-square and logistic regression analysis? It’s not clear how did you get this OR. | Authors acknowledge your valuable comments. Thank you.  The goodness of fit in logistic regression attempts to get at how well a model fits the data. To achieve goodness of fit of the regression model, variables in bivariate analysis whose p values were closer to Zero should be included in the multivariate analysis. This would further control confounding variables.  Previous studies[1–3], some of which have been published in PlosOne have used this approach to achieve goodness of fit and also to control for confounding variables.  The statement has been revised with clarity and now reads;  “Finally, all variables with p values less than 0.25 in the chi-square analysis were included in the logistic regression model, allowing all potentially important predictive factors to be included in the modified model and to control for confounding variables. Therefore, all variables with p values greater than 0.25 were not considered in the logistic regression models. Literature suggests that p values less than 0.3 or p value closer to zero (0) gives a desirable outcome of the goodness of a logistic regression model (21, 22). The benchmark p value of ≤0.25 was selected because of its closeness to zero (0) thus enabling an inclusion of all potentially relevant predictive variables in the adjusted model”  The revised text can be found on **Page 9 lines 183-191** |
| **Results** |  |
| The data should be presented in a clear way. <25 years…It’s not clear who the comparison group here and why the authors divided them in this way. For religion and education, there were multiple categories and it’s not clear what is the comparison group when they presented Christians (334, 88.6%) [vaccine acceptance; 67(20.1%) vs vaccine hesitancy and attained tertiary education (253, 67.1%) [vaccine acceptance; 65(25.7%) vs vaccine hesitancy. | Authors acknowledge your valuable comments. Thank you.  The Table 1 provides detailed results of the sociodemographic characteristics. However, the results narrations highlight important/significant findings of the study as presented. For example, the age <25 years comparator is ≥25 years (provided in Table 1). The other variables all have their comparators presented in the Table. Highlighting important findings has been adopted by other studies published by this journal. <https://doi.org/10.1371/journal.pone.0286186> and <https://doi.org/10.1371/journal.pone.0284643>  Authors categorized the age into <25 years and ≥25 years to have binary or dichotomous variables for the purpose of; (i) simplicity, (ii) ability to differentiate categories, and (iii) usefulness in the logistic regression and overall effective communication of the research findings.  **Page 10 Line 196-20**5 |
| Table 4 should be formatted in a professional way.  Table 5: What is COR?  Why did you exclude religion from the model.  Why was education categorised into binary? What do you mean by basic education?  When you conduct a multivariate logistic regression, you need to present the adjusted odds ratio, not the crude ratio. | Authors acknowledge your valuable comments. Thank you.  The Table 4 has been reformatted as suggested. The revised table can be found on **Page 17, Line 284**  The abbreviation “COR” was used to refer to crude odds ratio. It has now been defined in the manuscript and can be found on **Page 20 Line 314**    As indicated, the benchmark for the selection of variables for the logistic regression was p≤0.25; Religion had a p-value (p= 0.384) greater than the p≤ 0.25, and therefore did not meet the inclusion criteria for the logistic regression model development. Hence, it was excluded.  The educational categorizations were done based on Ghana’s educational system, where “basic education” is used to refer to six years of elementary education and three years of junior secondary/high education. Authors have subsequently defined the term “basic education” in the manuscript.  With the multivariate logistic regression analysis, authors presented, first the findings of the crude odds ratio and second, the adjusted odds ratio.  This was done to have a comprehensive narration of the analysis. The report of the crude odds ratios offers an initial understanding, while adjusted odds ratios account for potential confounding variables, control bias, enhance validity, and provide a more accurate estimate of the true association between the exposure and the outcome.  However, the discussion of the results was based on significant findings from the adjusted odds ratio. |
| **Discussion** |  |
| Lines 254-255 “The purpose of this study was to assess COVID-19 vaccine acceptance among urban dwellers in 255 Cape Coast, Central Region, Ghana” repetition of information. | Authors acknowledge your valuable comments.  The text have been deleted in the main manuscript. |
| Lines 347-349, “Age ≥25 years, female, educational level SHS and above, being employed, and hearing of new vaccine had a significant influence on COVID-19 vaccine acceptance”- repetition of information. | Authors acknowledge your valuable comments.  Authors acknowledge the importance of avoiding repetition of results in the discussion.  However, repeating the results in the discussion section was done to reinforce key findings, provide context and to guide the flow of the discussion. |
