# Supplementary material for: Coronavirus disease 2019 (COVID-19) vaccine acceptability in Ghana: An urban-based population study
Source: PLoS One. 2025 Mar 18;20(3):e0319798. doi: 10.1371/journal.pone.0319798 (PMC11918374; doi:10.1371/journal.pone.0319798)
Supplement: Attachment — Submitted filename: Mahvash Ansari_Review Commenting Document.docx [file pone.0319798.s005.docx]

| **Line Numbers of referenced text**  **(i.e. 29-43)** | **Referenced Text** | **Comment** | **Suggested Action (remove text, change text, add text, relocate text)** | **Rationale** |
| --- | --- | --- | --- | --- |
| 89 | However, [19] | Name of the author or study may be added | Add Text | To increase clarity |
| 231 | Vaccine acceptance was significant with knowledge of vaccination giving immunity | Vaccine acceptance was significant **among those** with knowledge of vaccination giving immunity | Add Text | To increase clarity |
| 244 | social media 73.50% (277/377), | **Social Media,** since all words start from uppercase | Add Text | Formatting |
| 389 | level from SHS and above, being employed, and hearing of new vaccine influenced on COVID-19 vaccine | level from SHS and above, being employed, and **information about** new **vaccines** influenced on COVID-19 vaccine acceptance. | Change Text | To increase clarity |
| 405 | Despite these limitations, the study can stillprovide valuable insights for public health interventions in Cape | Despite these limitations, the study can **still provide** valuable insights for public health interventions in Cape | Add Text | Formatting |
