# Supplementary material for: Coronavirus disease 2019 (COVID-19) vaccine acceptability in Ghana: An urban-based population study
Source: PLoS One. 2025 Mar 18;20(3):e0319798. doi: 10.1371/journal.pone.0319798 (PMC11918374; doi:10.1371/journal.pone.0319798)
Supplement: Attachment — Submitted filename: Responses to Reviewers_NM2024.07.30.docx [file pone.0319798.s004.docx]

Response to comments

| Reviewer #2:  The authors reported mutlistage cluster sampling however the sample size calculation provided in the methods section is not adjusted for the multistage clustering. Normally, the sample size is multiplied by the design effect to get the required power for the multistage cluster surveys. The given sample size is only based on simple random sampling and hence not adequate or underpowered. At this stage, the authors can only highlight this as an important limitation in the discussion section.  In addition, the authors have not adequately described the sample size calculation. Assumptions e.g., what proportion of COVID-19 vaccine hesitancy and acceptance was assumed to calculate the given sample size. | Thank you for your valuable comments and suggestions.  To estimate the sample size, the authors used a finite sample size estimator. This is because the study was a population-based study where the size of the population is known. Based on the sample size formula used, the design effect was not considered in arriving at the appropriate sample size which is a limitation to this study. We however acknowledged this shortfall under the strength and limitation section of this study on page 25 lines 409 to 410 and reads as follows; The sample size calculation provided in the methods section is not adjusted for the multistage clustering and hence not adequate or underpowered.    The proportion of COVID-19 vaccine hesitancy and acceptance used was assumed to be 50 percent. (**Page 8 Lines 152 to 156**) |
| --- | --- |
